# Novel fondaparinux protocol for anticoagulation therapy in adults with renal failure and suspected heparin-induced thrombocytopenia: a retrospective review of institutional protocol

**DOI:** 10.1186/s40360-023-00643-4

**Published:** 2023-01-13

**Authors:** Dania Ghaziri, Hassan Dehaini, Mayyas Msheik, Marwan Bahmad, Maya Zorkot, George Abi Saad

**Affiliations:** 1 Department of Pharmacy, American University of Beirut Medical Center, Beirut, Lebanon; 2 Departments of Vascular Surgery, Heart, Vascular and Thoracic Institute, Cleveland Clinic Foundation, Cleveland, USA; 3 Department of Surgery, American University of Beirut Medical Center, Beirut, Lebanon

**Keywords:** Fondaparinux, Anticoagulation, Thrombocytopenia, Renal failure, dialysis

## Abstract

**Introduction:**

The literature recommends against the use of fondaparinux in patients with kidney failure and dialysis as it may, with repeated dosing, accumulate and put patients at risk of bleeding. The management of patients with thrombosis in the presence of heparin-induced thrombocytopenia HIT requires the introduction of an alternative anticoagulant like bivalirudin or argatroban. When these drugs are not available, fondaparinux, remains the only alternative. In similar scenarios, there are few studies addressing how to administer it.

**Methods:**

We developed a protocol for fondaparinux in patients with renal failure where pharmacokinetic parameters are altered, and levels changed only after hemodialysis or in cases of residual renal activity. Patients received a full first dose except for high risk of bleeding. We targeted a peak anti-factor Xa activity level of 0.6–1.3 units/ml and changed the subsequent dose accordingly. Furthermore, we monitored the patients for signs of bleeding, a drop in hemoglobin level, or clinical signs of thrombosis.

**Discussion:**

We described 10 patients with kidney failure and suspected HIT taking fondaparinux. All the patients achieved therapeutic anti-factor Xa activity levels. However, one developed new-onset venous thromboembolism (VTE) despite therapeutic anti-factor Xa levels. Another patient experienced a bleeding episode. We believe that these two patients developed complications due to their medical conditions rather than the use of fondaparinux.

**Conclusion:**

Fondaparinux can be safely used in kidney failure using our protocol. However, despite its safety profile and relative success, this case series was small. More robust studies need to be conducted prior to drawing conclusions.

**Supplementary Information:**

The online version contains supplementary material available at 10.1186/s40360-023-00643-4.

## Plain Language Summary

New Fondaparinux Protocol to Reduce the Risk of Blood Thickening and Blood Clots Formation in Adults with Kidney Disease and Heparin-induced Thrombocytopenia (drop in platelets after the use of heparin): A Test Study.

Fondaparinux is a drug used to treat patients suffering from thrombosis (clot in blood) and prevent vessels occlusions. When patients have kidney disease, the ideal treatment for thrombosis would be heparin; and, in case of Heparin Induced Thrombocytopenia (HIT), an unexpected drop in platelets after the use of heparin, the ideal treatment would be argatroban or bivalirudin. Fondaparinux can be used for HIT. However, studies recommend against its use in kidney disease as it might accumulate and cause bleeding.

We were put in a challenging situation where we had patients with life-threatening thrombosis, kidney disease, HIT and unavailability of both argatroban and bivalirudin. Our only option was fondaparinux. We had to devise a safe and efficient protocol. The starting dose was the one used had the patient had a normal kidney function. Then, anti-Factor Xa activity was regularly measured with the target level 0.6-1.3units/ml 4 h after a dose. The dose was individualized, changed based on the Factor Xa activity result, the risk of bleeding or thrombosis, the overall kidney function and the need for dialysis.

Our protocol was tested on 10 patients. All our patients could reach the target and safe Factor Xa activity. We had 2 exceptions. The first had a clotting event despite having therapeutic Factor Xa activity and the second was a very sick cancer patient who was bleeding despite skipping many doses of fondaparinux. We consider that these 2 cases developed complications due to their medical conditions rather than the use of fondaparinux.

We concluded that fondaparinux can be safely used in patients with kidney disease, granted that Factor Xa activity is measured, the risk of bleeding is weighed to the risk of thrombosis and the dose is individualized. However, our sample size is small and further studies with a larger number of patients are needed to draw a conclusion.

**Supplementary Information:**

The online version contains supplementary material available at 10.1186/s40360-023-00643-4.

## Introduction

Patients with renal failure are at increased risk of bleeding when treated with anticoagulants [1]. Therefore, these patients are commonly treated with unfractionated heparin (UFH) in hospital settings; its short therapeutic half-life (which is not prolonged with renal failure) and complete reversal with protamine permit easier control of bleeding episodes [2]. However, the use of UFH carries multiple risks, one of which is heparin-induced thrombocytopenia (HIT). HIT, particularly type 2, is an autoimmune-like reaction in which antibodies bind to platelet factor 4 (PF4)/heparin complexes that activate platelets via their FcγIIa receptors, resulting in thrombocytopenia, hypercoagulability, and greatly increased risk of arterial or venous thrombosis [3]. The potential complications of HIT type 2 are numerous, including venous thromboembolism (VTE), stroke (arterial, venous), myocardial infarction, skin necrosis (including at heparin injection sites), adrenal hemorrhagic necrosis (which if bilateral can cause acute and chronic adrenal failure), limb amputation (secondary to arterial thrombosis or venous limb gangrene), and death [4, 5]. Consequently, when this entity is confirmed or suspected, alternative anticoagulants are used, preferably direct thrombin inhibitors, such as bivalirudin or argatroban [6]. Unfortunately, these drugs are unavailable in low-income countries like ours (Lebanon), rendering fondaparinux the only accepted alternative (accepted by the consensus guidelines of the American Society of Hematology ) treatment for this life-threatening condition [7] Additionally, switching to direct oral anticoagulants (DOACs) is not always safe, particularly in a patient population with high acuity and critical illness such as ours where short-acting anticoagulants are generally preferred and where physiologic changes and other changes brought on by using vaso-active drugs and narcotics, alter gastric perfusion and motility, making the anticoagulant effect of DOACs unpredictable [8]. This is more important when UFH is initially prescribed to treat other conditions, such as pulmonary embolism (PE), VTE, or atrial fibrillation (AF), where omitting anticoagulation is also life-threatening.

Fondaparinux is a synthetic pentasaccharide with biological activity based on the selective, antithrombin-mediated inhibition of activated factor X (factor Xa). Owing to its synthetic origin and small molecular structure, it has low immunogenicity and infrequent cross-reactivity with HIT antibodies, and thus can be safely used in the case of HIT type 2 [9]. Nevertheless, it is generally not recommended to use fondaparinux in patients with renal failure because of the inherent risk of bleeding in these patients, the absence of a reversal agent, and the accumulation of fondaparinux in the body even in patients receiving intermittent hemodialysis (HD) [10, 11]. Therefore, the current use of fondaparinux for renal failure is individualized and based on thrombosis and bleeding risks. This is difficult, given the lack of guidelines and paucity of research on this subject. Consequently, despite these limitations, we developed a dosing protocol for fondaparinux in patients with renal failure, suspected HIT type 2, and requiring therapeutic anticoagulation, which we described in this case series.

We faced a major challenge in determining the appropriate fondaparinux dose and anti-factor Xa monitoring because we had to use the anti-factor Xa curves specific to low molecular weight heparin (LMWH), the only available assay in our laboratories. It is also worth mentioning that, in our facility, we did not carry out any tests to measure the level of fondaparinux or anti-PF4/heparin antibodies.

## Methods

We built an internal protocol based on the assumption that pharmacokinetic parameters such as steady-state, peak, and trough are altered in patients with renal insufficiency. For patients on HD, we relied on the fact that the levels would only change after HD because of the very low elimination before dialysis and the low molecular weight of fondaparinux (1.7 kDa), which can be eliminated through the high-flux dialysis membrane. Therefore, for dialysis patients, we redose only after dialysis.

Using the LMWH assay, the only available assay in our facility, and with the impossibility to calibrate this assay to the fondaparinux standard curve, we targeted a conservative therapeutic level of anti-factor Xa (anti-Xa) activity of 0.6–1.3 Units/ml, which is 20% [12] above the usual therapeutic target for enoxaparin (0.5-1Units/ml [13]).

During the administration of fondaparinux, we monitored peak anti-Factor Xa levels 4 h after a dose,and followed up for signs of bleeding on physical examination, drop in hemoglobin level, and clinical signs of thrombosis. To assess the risk of bleeding, we measured daily platelet count, international normalized ratio (INR), and partial thromboplastin time (PTT). Finally, we performed a daily clinical assessment of bleeding risk.

The first dose was a full therapeutic dose that would have been administered to the patient with normal kidney function. This dose was administered by subcutaneous injection and determined based on the weight of the patient as follows:

- Weight ≥ 100 kg: 10 mg.
- Weight 50–99 kg: 7.5 mg.
- Weight < 50 kg: 5 mg.

However, the choice of the first dose was based on clinical judgment, which weighed the risk of thrombosis with that of bleeding. Furthermore, it took into consideration the presence of any residual renal activity that favored a full first dose. A reduced first dose (e.g., 5 mg or even 2.5 mg instead of 7.5 mg) was given to patients with a high risk of bleeding.

Subsequent doses were given as follows:

Peak anti factor-Xa levels were measured 4 h after the first dose, and subsequent doses were dependent on this result, as follows:

- anti-factor Xa > 1.3 Units/ml: reduce dose by 2.5 mg.
- anti-factor Xa 0.6–1.3 Units/ml: maintain the same dose.
- anti-factor Xa < 0.6 Units/ml: increase dose by 2.5 mg.

Subsequent monitoring was performed as follows:

- Peak anti-factor Xa level 4 h after each dose for all patients. If the level is stable for more than three doses, there is no need to check the level after each dose, except if a change in renal function occurs. Random monitoring was performed every few days.
- For HD patients (re-dosing only after dialysis), only peak anti-factor Xa level at 4 h post-dose is needed. Additional random levels is taken only if needed for close monitoring.
- For non-HD patients with residual renal activity, an additional random level can be ordered to help decide on redosing.

To note that, despite the fact that our protocol mentions measuring “*PF4 antibodies for patients with high 4T scores*”, the suspicion of HIT was never confirmed by any laboratory testing (enzyme-linked immunosorbent assay ELISA or platelet serotonin-release assay SRA) of anti-PF4/heparin antibodies because these do not exist in our facility. Furthermore, due to various logistical and financial challenges, sending for antibody testing was not possible.

## Results

Ten patients with renal failure who required therapeutic anticoagulation with fondaparinux and suspected HIT type 2 because of thrombocytopenia with recent/ongoing exposure to UFH or LMWH were included in this case series (Table 1). None of the patients had thrombosis at the time of suspicion of HIT. The median 4T score was 5, indicating an intermediate risk of HIT. The median age of the patients was 74 years old (58.5, 81.75) and 60% of them were males. The median weight was 78 kg (68.25, 103); 3 patients had a weight above 100 kg, while the remaining patients had a weight between 50 and 100 kg. The reason for admission was variable among the 10 patients, but they all had cardiac or septic shock. Only 4 patients had undergone recent surgeries, but they shared several comorbidities, such as hypertension (60%), diabetes mellitus (DM, 60%), and coronary artery disease (50%). Other notable conditions include solid and hematologic malignancy, valvular heart disease, abdominal aortic aneurysm, autoimmune disease, chronic obstructive pulmonary disease, and glucose-6-phosphate dehydrogenase deficiency. In total, half of the patients were hemodialysis-dependent, one patient had acute kidney injury, and the remaining four had chronic kidney disease (CKD) stage 4.

**Table 1:** Summary of patient demographics

	Patients									
	1	2	3	4	5	6	7	8	9	10
Age (y)	72	76	87	54	89	76	60	80	47	64
Sex	M	M	F	M	M	F	F	F	M	M
Weight (kg)	101	115	69	109	70	66	82	83	74	60
LOS	35	31	36	81	55	10	18	11	35	20
RFA	Covid-19	Fournier gangrene	Pneumonia	Bowel perforation	UTI	Ischemic colitis	Bleeding pelvic tumor	VHD	Covid-19	Symptomatic thrombocytopenia
Comorbidities										
AAA			✘							
Autoimmune disease						✘				
CAD	✘	✘	✘		✘			✘		
COPD		✘								
DM	✘	✘		✘	✘	✘		✘		
G6PD deficiency									✘	
HTN	✘	✘	✘		✘	✘		✘		
Malignancy				✘			✘		✘	✘
VHD			✘					✘		
Renal function	HDD-ESRD	HDD-ESRD	CKD stage 4	HDD-ESRD	CKD stage 4	CKD stage 4	HDD-ESRD	CKD stage 4	AKI (CrCl 30 mL/min)	HDD-ESRD
Recent Surgery		Vascular	Cardiac				Oncologic	Cardiac		
Indication for anticoagulation	AF	AF	AF	VTE	AF	VTE	Acute limb ischemia	AF	VTE	VTE
4T score	4	5	4	5	5	4	5	5	4	5
First dose (mg)	5	7.5	5	7.5	5	2.5	7.5	2.5	7.5	5
First anti-Xa	0.60	0.38	0.42	0.60	0.60	0.39	0.22	0.22	1.07	0.79
# of doses needed to reach therapeutic level	1	2	2	1	1	2	-	2	1	1
Total days on Fondaparinux	5	10	20	7	15	14	2	4	11	10
Number of anti-Factor Xa measures	4	5	14	6	10	11	2	5	7	5
Signs of bleed									✘ chest tubes /pneumothorax	
Drop in platelets after the start of fondaparinux	increase	increase	increase	✘ moderate drop platelets 50–100 × 10 9 /L	✘ moderate drop platelets 50–100 × 10 9 /L	increase	stable	stable	✘ severe drop (malignancy) platelets less than 50 × 10 9 /L	✘ severe drop (malignancy) platelets less than 50 × 10 9 /L
New thrombosis				✘						

*AAA* Abdominal aortic aneurysm, *AF* Atrial fibrillation, *CAD* Coronary artery disease, *CKD* Chronic kidney disease, *COPD* Chronic obstructive pulmonary disease, *CrCl* Creatinine clearance, *DM* Diabetes mellitus, *G6PD* Glucose-6-phosphate dehydrogenase, *HDD-ESRD* Hemodialysis-dependent-end-stage renal disease, *HTN* Hypertension, *LOS* Length of stay, *RFA* Reason for admission, *UTI* Urinary tract infection, *VHD* Valvular heart disease, *VTE* Venous thromboembolism

The most common indication for initial anticoagulation was atrial fibrillation (50%), followed by venous thromboembolism (VTE, 40%), and acute limb ischemia (10%). Four patients were started on 7.5 mg; four patients received an initial dose of 5 mg, and 2 patients received an initial dose of 2.5 mg due to an elevated risk of bleeding. Half of the patients achieved a therapeutic anti-factor Xa level after the first dose, whereas the other half required repeated doses. None of our patients had a supratherapeutic level of anti-factor Xa, and all patients maintained a therapeutic level throughout their treatment except if intentionally avoided. Nevertheless, despite a therapeutic anti-factor Xa level, one patient developed thrombosis, manifested as a new-onset asymptomatic lower-limb thrombosis (VTE) that was found accidentally while performing a routine duplex scan of the lower extremities.

Following the start of fondaparinux therapy, platelet count increased (to less than double the lowest it reached, but without reaching back baseline) in 4 patients, while it remained stable in 2 others. However, it moderately dropped in 2 patients and severely dropped in 2 others; these two were believed to have progressive thrombocytopenia associated with malignancy. One experienced a bleeding episode in the setting of worsening thrombocytopenia that required platelet transfusion.

## Discussion

A few case reports described the use of therapeutic fondaparinux in kidney failure and dialysis patients [14, 15], and the available literature recommends against its use [10]. We were put in a challenging situation where we had to treat patients with severe life-threatening thromboembolic conditions, severe thrombocytopenia, or possible HIT type II, in the absence of local availability of direct thrombin inhibitors such as bivalirudin or argatroban [7]. Owing to their severe illness, risk of bleeding, or recent surgeries, it was impossible to treat them with DOACs. In fact, important physiologic alterations typical to ICU patients, and the addition of vaso-active or narcotic pain medications may cause DOACs to have unpredictable anticoagulant effects. This may be due to impaired gastric perfusion and motility, increased volume of distribution, reduced hepatic clearance (inhibition of enzymatic metabolism), and changes in renal function. Finally, our institution does not carry Apixaban, the only DOAC approved for severe renal failure and dialysis [8].

Our protocol was designed and built using the limited available data on fondaparinux dosing in renal failure/dialysis, and the few laboratory testing resources available in our facility relied on the clinical judgment of the medical team to weigh the risk of bleeding for each patient. Considerations such as complications of previous surgeries, coagulopathy, history of bleeding or any other bleeding risk, and the presence of residual renal activity were considered. In parallel, we relied on the availability of recombinant factor VIIa at our institution [7], which is appropriate for reversing the anticoagulant effect of fondaparinux in cases of bleeding [14].

Our experience using the protocol described in this study was successful. We succeeded in safely achieving therapeutic anti-Factor Xa levels without putting patients at risk of bleeding or thrombosis. However, there were two exceptions. The first (patient 4 in Table 1) was treated for deep venous thrombosis (DVT); this patient developed a new DVT in the right femoral vein despite achieving a therapeutic level of anti-factor Xa while on fondaparinux. The patient was admitted for bowel perforation and was known to have DM, bladder cancer, and hemodialysis-dependent end-stage renal disease, increasing his risk for thrombosis. The second exception was a patient with coronavirus disease − 19 (COVID-19), known to have diffuse large B cell lymphoma (DLBCL), who deteriorated into septic shock. He was bleeding from his chest tubes, despite skipping many doses of fondaparinux and reaching subtherapeutic levels of anti-factor Xa (0.3U/ml). We believe that these two patients developed complications due to their medical conditions rather than the use of fondaparinux. Moreover, two of our patients were started on 2.5 mg intentionally despite achieving a subtherapeutic initial level due to their elevated risk of bleeding.

Limitations of our study include the lack of serological confirmation of the diagnosis of HIT; thus, it is uncertain to what extent our study’s findings can be generalized to a HIT patient population. Other limitations include the small number of patients we studied and the unavailability of an anti-factor Xa assay using a fondaparinux standard curve (however, we used a LMWH standard curve with an appropriate adjustment in the target therapeutic range).

## Conclusion

Our experience using the newly devised fondaparinux protocol for kidney failure, with or without dialysis, was successfully introduced in our tertiary institution. We did not experience any complications related to the use of the protocol but rather because of the patients’ morbid conditions. This is more important because the protocol safely targets a variety of critically ill medical and surgical patients with a wide range of thrombotic and bleeding risks. Despite the safety profile and relative success of the protocol, there were many limitations, mainly the paucity of laboratory resources and small number of patients. A more robust study design with a larger number of patients and, in comparison, other alternatives need to be conducted prior to endorsing such a protocol. Furthermore, to properly monitor our patients, we recommend the development of a local fondaparinux-specific anti–factor Xa assay.

## Supplementary Information

**Additional file 1.**

## Data Availability

The datasets generated and/or analyzed during the current study are available from the corresponding author on reasonable request.

## Declarations

The experimental protocol was approved by our institutional review board IRB at the American University of Beirut Medical Center (AUBMC) and all methods were carried out in accordance with relevant evidence, guidelines and regulations.
