# Supplementary material for: Novel fondaparinux protocol for anticoagulation therapy in adults with renal failure and suspected heparin-induced thrombocytopenia: a retrospective review of institutional protocol
Source: BMC Pharmacol Toxicol. 2023 Jan 13;24:2. doi: 10.1186/s40360-023-00643-4 (PMC9837750; doi:10.1186/s40360-023-00643-4)
Supplement: Supplementary file 1 — Additional file 1. [file 40360_2023_643_MOESM1_ESM.docx]

|  | Identification  label | | |
| --- | --- | --- | --- |
| **Adult Therapeutic Fondaparinux in Kidney Failure/Dialysis patients –Order Set** |  |  |  |
| **Last Name: ______________________________________________**  **First and Middle Name: __________________________________**  **Patient Number: _________________________________________**  **Date of Birth: ________________________ Age: _______________**  **Gender: 🞏 Male 🞏 Female**  **Admission Date: _________________________________________**  **Admitting Physician: _____________________________________** | **Unit: ______________________________________________**  **Weight: _________________ Height: __________________**  **Expanded Precautions:** 🞏 None 🞏 Airborne 🞏 Droplet  🞏 Contact 🞏 Contact Plus  **Other Precautions: _________________________________** | | |
|  | **Allergy □ No □Yes (specify reaction):** | | |
| **The following abbreviations may not be used to document patient care: U IU QD QOD .X mg X.0 mg MS MSO_4_ MgSO_4_ CC µg mcg**  ADDRESOGRAPH  **🗹 Check the Applicable Order** | | | |
| Laboratory tests:  **🗹** INR daily  **🗹** PT, PTT daily  **🗹** Platelets daily  🞏 CBC differentials daily  🞏 Chem 9 daily  **🗹** Fibrinogen weekly  **🗹** D-Dimer weekly  🞏 US upper extremities for patients with CVCs to screen for occult thrombosis once  🞏 US lower extremities for all patients to screen for occult thrombosis once  🞏 PF4 antibodies for patients with high 4T scores  Others: | | **Nurse’s Name and Signature** | **Time Noted** |
| **Goal:**  **🗹** Maintain anti-Factor Xa activity between 0.6-1.2mg/l  **🗹** Monitor for signs of bleeding or filter clotting during dialysis  **🗹** Report any bleeding or clotting during dialysis to modify regimen accordingly  **🗹** For patients on dialysis, lock catheter with hypertonic trisodium citrate 4% | |  |  |
| Dosing:  **🗹** Dosing depends on an assessment of the patient weighing the risk of bleeding (elevated INR, very low platelets count versus need for anticoagulation.  **🗹** Reassess dosing with every improvement or deterioration of kidney function  **🗹** Use **clinical judgment** when deciding on re-dosing if residual renal activity, a new risk of bleeding or, accumulation  **🗹** Can order an extra random level when needed  **🗹** Can skip a dose when needed  **Starting dose**  **Starting dose: Select one of the following (give after dialysis if on dialysis)**  🞏 10mg if weight greater than 100kg  🞏 7.5mg if weight between 50kg and 100kg  🞏 5mg if weight less than 50kg  **Reduced starting dose if the risk of bleeding (give after dialysis if the patient is on dialysis)**  🞏 7.5mg 🞏 5mg 🞏 2.5mg    **Subsequent doses**  **Re-dosing dialysis patients**  **🗹** Re-dose only after dialysis on days of dialysis  **🗹** Measure anti-Factor Xa activity levels 4 hours after the dose  🞏 anti-Factor Xa activity level > 1.3 mg/l: reduce next post dialysis dose by 2.5mg  🞏 anti-Factor Xa activity If level 0.6-1.3mg/l: keep same dose post next dialysis  🞏 anti-Factor Xa activity If level < 0.6 mg/l: increase next post dialysis dose by 2.5mg  **Re-dosing non-dialysis patients**  🞏 Re-dose every 24 hours  🞏 Re-dose based on daily assessment  **🗹** Measure Anti-Factor Xa activity 4 hours after the dose  🞏 anti-Factor Xa activity level > 1.3 mg/l:  🞏 reduce the next day’s dose by 2.5mg 🞏 skip the next day’s dose  🞏 anti-Factor Xa activity level 0.6-1.3 mg/l: keep same dose  🞏 anti-Factor Xa activity level < 0.6 mg/l: increase next dose by 2.5mg | |  |  |

**MD Name: _______________________________________ Signature: ____________________________________**

**Date: ____________________________________________Time: _____________ Pager Number: _____________**

Original: Medical Record **~~ FAX OR SCAN TO PHARMACY ~~**

Copy: Pharmacy

1/2 Stock #
